# Dermatological Ideas

**Published:** 1908-01

**Authors:** 


### DERMATOLOGICAL IDEAS.

---

Aronstam formulates ten practical ideas for the management of skin diseases, in substance as follows:

1. Calomel, sodium salicylate and small doses of belladonna will benefit the most intractable cases of urticaria, even after other internal and external remedies have failed.

2. In acute erythematous eczema of the extremities, where the subjective symptoms are very pronounced and annoying, the application of a dilute solution of the adrenal principle to the lesions will produce a rapid blanching of the parts and ameliorate the intolerable itching. After the acute features have subsided, ordinary Lassar's paste, with or without ichthyol, wherein a small amount of adrenalin solution has been incorporated, will prove curative.

3. Bakers yeast administered in one-half dram doses 3 hours after meals, just at time when the food has undergone chylification in the small intestines, is occasionally of signal service in furuncles. It is especially indicated when indican is detected in the urine of patients afflicted with repeated outbreaks of this affection.

4. The little chamois-yellow papules and macules on the lower lids at the canthi and sometimes on the dorsal surfaces of the hands and forearms are exanthematous in nature, and indicate either renal lesions, or affections of the pancreas and liver. They have also been met in patients suffering from glycosuria. An examination of the urine of all such patients is therefore imperative.

5. Iodide of potash may at times produce an angio-neurotic edema of the eyes and face. This is a serious drawback for its exhibition in syphilis. It can, however, be avoided by combination with belladonna or hyosaymus.

6. Remember to forbid including the p's— pickles, porridge, pork, pastry and potatoes—in the dietary of patients suffering from cutaneous diseases.

7. Induced hyperemia is of paramount usefulness in combating indolent and indurated acne. The Bier's apparatus must, however, be kept on the individual lesions for at least five to ten minutes before it is withdrawn, shorter periods being of little avail.

8. Fatty applications are, as a rule, not well borne on the scalp or other hairy parts of the body. When employed, the hair must be clipped short. In girls, where the cutting of hair is refused, we must have recourse to lotions, preferably aqueous or alcoholic, which invariably act more favorably than unctuous preparations.

9. Never use aqueous lotions in acute vesicular or erythematous eczema, since water nearly always irritates and intensifies the pathologic process. Protection is the great indication, and pastes or powders should be applied.

10. Pityriasis rosea closely resembles ringworm of the body. The former, however, presents larger crescentric and circinate lesions than the latter. The macules look yellow, do not exhibit an inflammatory zone nor vesiculation at its margin; they are but slightly scaly, and, as a rule, invade the trunk in preference. They are non-parasitic, and disappear under different medication within a fortnight.—Medical Fortnightly.

